# Therapist-guided, Internet-delivered cognitive behaviour therapy for adolescents with body dysmorphic disorder: A feasibility trial with long-term follow-up

**DOI:** 10.1016/j.invent.2023.100688

**Published:** 2023-11-16

**Authors:** Daniel Rautio, Per Andrén, Martina Gumpert, Maral Jolstedt, Amita Jassi, Georgina Krebs, Markus Jansson-Fröjmark, Tobias Lundgren, Eva Serlachius, David Mataix-Cols, Lorena Fernández de la Cruz

**Affiliations:** a Centre for Psychiatry Research, Department of Clinical Neuroscience, Karolinska Institutet, Stockholm, Sweden; b Stockholm Health Care Services, Region Stockholm, Stockholm, Sweden; c Department of Clinical Sciences, Lund University, Lund, Sweden; d National and Specialist OCD, BDD, and Related Disorders Clinic for Young People, South London and Maudsley NHS Foundation Trust, London, England, United Kingdom; e University College London, Research Department of Clinical, Educational and Health Psychology, London, England, United Kingdom

**Keywords:** Body dysmorphic disorder, Dysmorphophobia, Evidence-based interventions, Internet-based treatment, Cognitive behaviour therapy, Treatment outcomes, Adolescents

## Abstract

Body dysmorphic disorder (BDD) is a prevalent and impairing psychiatric condition that typically debuts in adolescence and is associated with risky behaviours. The disorder can be effectively treated with cognitive behaviour therapy (CBT). However, CBT for BDD is seldom available primarily due to a shortage of trained therapists. Internet-delivered CBT (ICBT) can be a way to increase treatment availability. The aim of this feasibility trial was to evaluate the feasibility, safety, and preliminary efficacy of a CBT protocol for adolescents with BDD, adapted to be delivered over the Internet with minimal therapist support. A total of 20 participants (12–17-year-olds) meeting criteria for BDD were recruited nationally to a specialist outpatient clinic in Stockholm, Sweden. One participant withdrew consent and their data could not be analysed. Nineteen participants were offered 12 modules of therapist-guided ICBT for BDD and were followed up to 12 months post-treatment. Preliminary efficacy was measured at the a priori primary endpoint (3-month follow-up) and at the 12-month follow-up with the clinician-rated Yale-Brown Obsessive Compulsive Scale Modified for BDD for Adolescents. The treatment was rated as both credible and satisfactory and was associated with a large and statistically significant reduction in BDD symptom severity (*d* = 2.94). The proportion of participants classified as responders at the primary endpoint was 73.7%, and the proportion of full or partial remitters was 63.2%. The average therapist support time was 8 min per participant per week. Treatment gains continued to accrue up to the 12-month follow-up. Two participants attempted suicide and another two reported non-suicidal self-injuries during the study period. ICBT with minimal therapist support is a feasible, potentially efficacious, and durable treatment for adolescents with BDD. Risky behaviours typical of this patient group should be carefully monitored during treatment.

## Highlights

• CBT for adolescents with body dysmorphic disorder (BDD) is seldom available.
• Internet-delivered CBT (ICBT) can be a way to increase treatment availability.
• ICBT is a feasible treatment option for adolescents with BDD.
• ICBT may be both efficacious and cost-effective, with durable symptom reductions.
• Suicidal behaviours need to be carefully monitored during treatment.

## 1 Introduction

Body dysmorphic disorder (BDD) is a mental disorder characterised by a preoccupation with perceived defects or flaws in physical appearance, leading to time-consuming repetitive behaviours (i.e., mirror checking, camouflaging), marked avoidance, and significant distress and impairment (American Psychiatric Association, 2013). The prevalence of the disorder is approximately 2% in community samples of both adolescents and adults (Enander et al., 2018; Schneider et al., 2017; Veale et al., 2016). BDD usually emerges at an early age, with a reported mean age of onset around 12 to 16 years (Bjornsson et al., 2013; Phillips et al., 2006; Rautio et al., 2022). In young people, BDD is associated with several important risky behaviours, including an elevated risk of suicidal ideation and suicide attempts, self-harm behaviours, desire for unhelpful cosmetic procedures, risky sexual behaviours, and school dropout (Krebs et al., 2017; Phillips et al., 2006; Rautio et al., 2022).

Cognitive behaviour therapy (CBT) is the first-line treatment for both adolescents and adults with BDD (Harrison et al., 2016; NICE, 2005), although the evidence supporting its efficacy in young people is still developing (Greenberg et al., 2016; Krebs et al., 2012; Mataix-Cols et al., 2015; Rautio et al., 2022). The benefit of CBT for adolescent BDD seems durable at least up to one year after treatment (Krebs et al., 2017; Rautio et al., 2022). However, CBT for BDD is a highly specialised treatment that is often unavailable due to lack of trained therapists and large geographical distances to a limited number of centres that offer these interventions (Comer and Barlow, 2014). Hence, alternative ways of making CBT more available should be considered.

One such alternative modality is therapist-guided, Internet-delivered CBT (ICBT) (Andersson et al., 2019). In ICBT, the participants follow an interactive online treatment manual and receive support and guidance from a trained professional through online communication. ICBT requires less therapist time, eliminates geographical distances, and participants do not need to schedule appointments during their school day and can access the treatment materials at any chosen time. ICBT has shown to be both efficacious and cost-effective for a wide range of somatic and mental disorders in children, adolescents, and adults (Hedman-Lagerlöf et al., 2023; Vigerland et al., 2016). ICBT can also provide low threshold access to mental health services and be used as a cost-effective tool for early detection and intervention for various psychiatric conditions (Seiferth et al., 2023). Additionally, ICBT can be offered in a stepwise fashion, with individuals who fail to benefit from ICBT having the possibility to be offered additional in-person CBT (Jolstedt et al., 2021). An ICBT program for adults with BDD, called BDD-NET, was successfully evaluated in a pilot study (Enander et al., 2014) and in a randomised controlled trial (RCT) (Enander et al., 2016), with response rates in line with traditional face-to-face CBT (Harrison et al., 2016) and sustained treatment effects up to a two-year follow-up (Enander et al., 2019). Recently, a real-world implementation study of BDD-NET in the Swedish public health system has also demonstrated excellent results and reduced waiting times to receive treatment at the clinic (Lundström et al., 2023). By contrast, research on ICBT in adolescents with BDD is, much more limited (Hartmann et al., 2021).

In this feasibility trial, we adapted a previously developed (Mataix-Cols et al., 2015; Rautio et al., 2022) face-to-face CBT treatment manual for adolescents with BDD into a guided ICBT format and evaluated its feasibility, safety, preliminary efficacy, and durability of gains.

## 2 Methods

The Swedish Ethical Review Authority approved the study (reference number 2021-01942), and informed consent was provided by all participants and their parents/legal guardians. The trial was pre-registered at ClinicalTrials.gov (registration ID: NCT05078320).

### 2.1 Setting and procedures

All patients referred to the obsessive-compulsive disorder (OCD) and related disorders specialist clinic for children and adolescents in Stockholm, Sweden were asked to participate in the study. The clinic accepts referrals from all over the country. The trial was also advertised to clinics across Sweden, to interest organisations (e.g., the Swedish OCD organisation), and directly to the public via the official website of the Stockholm child and adolescent mental health services. Paid advertisements were also distributed via Facebook.

To be eligible to participate in the study, participants had to be 12 to 17 years of age, have a primary DSM-5 diagnosis of BDD (American Psychiatric Association, 2013), a score on the Yale-Brown Obsessive Compulsive Scale Modified for BDD for Adolescents (BDD-YBOCS-A) (Phillips et al., 1997) ≥24, at least one available parent/caregiver to support the adolescent, and access to a computer with Internet connection and a mobile phone. Exclusion criteria included: previous CBT for BDD (8 or more sessions) within the past 12 months, ongoing psychological treatment for BDD, adjustment or change of any selective serotonin reuptake inhibitors (SSRI) within 6 weeks prior to enrolment, a diagnosis of organic brain disorder, intellectual disability or a psychiatric disorder that could interfere with the treatment (e.g., psychosis, eating disorder), immediate risk to self or others requiring urgent medical attention (such as current risk of suicide or self-injurious behaviours) or child and caregiver not being able to communicate in Swedish.

Initial assessments consisted of a 2-h session where participants completed a series of clinical interviews, including a semi-structured interview based on the Structured Clinical Interview for DSM-IV (SCID-I) (American Psychiatric Association, 2018), adapted by our research group to align with the more recent DSM-5 criteria for BDD and other obsessive-compulsive related disorders, and the Mini International Neuropsychiatric Interview for Children (Sheehan et al., 1998) to assess other comorbidities. Socio-demographic information, detailed data on school attendance, and current desire for and/or whether participants had undergone any cosmetic/surgical procedure related to their BDD concerns were also collected. Finally, participants were screened for substance use using the CRAFFT, a 10-item self-reported screening instrument designed to identify substance use (including nicotine, alcohol, and other drugs) (Knight et al., 1999). Initial assessments were conducted at the clinic (*n* = 7) or via video conference (*n* = 12) by an experienced clinical psychologist (DR). Assessments were then discussed in a multidisciplinary team including clinical psychologists and a child and adolescent psychiatrist.

### 2.2 Treatment

The treatment was therapist-guided ICBT, involving both the adolescent and at least one parent/caregiver. The treatment consisted of two separate sets of 12 modules (one for the adolescent and one for the parent/caregiver), each with separate logins, delivered over a maximum of 14 weeks. In certain circumstances (e.g., illness or holidays), participants could pause their therapist support for one or two weeks. A new module was made available to the families every week, regardless of whether they had completed the preceding modules. After the active treatment period (12 to 14 weeks), the families could continue to access all treatment modules, without therapist support, for the whole duration of the follow-up period (12 months).

During treatment, participants had regular asynchronous contact within the treatment platform with a trained therapist (DR) who provided feedback, answered questions, and reminded to complete the next module, if required. Communication was possible via text messages within the treatment platform, and the therapist replied to messages within 48 h. Phone calls were possible if the participant or the therapist felt they were needed but were generally kept to a minimum. These communications were not structured.

The CBT treatment manual, originally developed at King's College London and evaluated in the only RCT for adolescents with BDD to date (Mataix-Cols et al., 2015) and further improved and evaluated in a naturalistic study (Rautio et al., 2022), was adapted from face-to-face into an online format. Besides the difference in the format of delivery, the content of the online version is virtually identical to that of the face-to-face manual. To facilitate the delivery, the online version includes illustrations, case examples, and videos explaining the treatment content. To improve user experience and to create a positive and reinforcing treatment environment, we involved young people with BDD and their caregivers in this adaptation process (Seiferth et al., 2023). The main goal of the treatment is to help the young person to stop avoiding anxiety-provoking situations (e.g., going to school or other social situations) by undertaking exposure tasks and to stop doing unhelpful repetitive behaviours and rituals (i.e., excessive mirror-checking, camouflaging, excessive use of make-up), known as response prevention. All modules also contained homework tasks to be completed between modules, which mainly consisted of exposure and response prevention (ERP) tasks based on the young person's individual goals. Parents had their own separate logins and modules and were encouraged to be involved throughout the treatment. Parental modules included psychoeducation, information on family accommodation (Jassi et al., 2020a) and how to reduce it, and strategies to assist their child in the different ERP tasks. An overview of the ICBT modules is shown in **Supplementary Table 1**. Example screenshots from the treatment are presented in **Supplementary Fig. 1**.

After finishing ICBT, all participants were offered five follow-up assessment appointments (1, 2, 3, 6, and 12 months after treatment) to measure symptom severity and evaluate risk and need for additional treatment. The a priori primary endpoint of the study was the 3-month follow-up. Due to ethical concerns, if participants were classified as non-responders (see 2.3.3 below) at any of the previous assessment points, they were offered booster sessions consisting of fortnightly one-hour video sessions with an experienced therapist (DR). Additionally, participants that were classified as non-responders at the primary endpoint were offered additional face-to-face treatment at the clinic.

### 2.3 Outcome measures

#### 2.3.1 Feasibility measures

To assess feasibility, several variables were collected at different time points. *Participant retention, treatment completion, and acceptability* were evaluated by investigating recruitment rates to determine whether enough number of potential participants met the pre-established eligibility criteria and whether eligible participants accepted the offered online format. Further, number of completed modules and attrition rates throughout the study period were also collected. We also measured participant drop-out rates and reasons for dropping-out, and whether they received any treatment elsewhere. This was done by interviewing participants and caregivers if a drop-out occurred and by recording concomitant interventions at all time points. To assess the *participant's adherence* to the treatment, the internet intervention Patient Adherence Scale (iiPAS) (Lenhard et al., 2019) was used. The iiPAS consists of 5 items, each rated on a scale from 0 to 4, including ratings of engagement in homework, communication with the therapist, and login frequency. To assess *treatment adherence*, we evaluated how many modules the participants and their caregivers completed during the therapist-guided treatment time (14 weeks), how often they logged in to the platform, and how often they sent and received messages. *Treatment credibility* was measured with a short self- and parent-reported questionnaire created by the research team and used in previous ICBT trials (Andrén et al., 2019). The questionnaire consists of 3 items asking how well the treatment suits young people with BDD symptoms, how much improvement they expect from the treatment, and how motivated they feel to work with the treatment. Each item is scored on a 0 to 4 Likert scale. *Treatment satisfaction* was measured with the Treatment Satisfaction Questionnaire, a 7-item self- and parent-reported measure created by our research team, also used in previous ICBT trials (Andrén et al., 2019).

#### 2.3.2 Safety measures

The modified version of the Negative Effects Questionnaire (Rozental et al., 2016) was filled in by the parents, with input from the adolescent, at mid-treatment, post-treatment, and at the 3-month follow-up. The scale consists of 17 short items relating to common adverse events (e.g., headaches, anxiety, sleeping problems).

Additionally, every three weeks during the active phase of the treatment, participants and their caregivers completed the 13-item Short Mood and Feelings Questionnaire, Child (SMFQ-C) and Parent Versions (SMFQ-P) (Rhew et al., 2010), including an additional item aimed at assessing suicide risk during the past week*.* A score >13 on the SMFQ or a score of 2 or 3 on the suicide item automatically raised a flag in the system to directly notify members of the research team to follow this up (via telephone) with the participant's family.

Non-suicidal self-injurious behaviours and suicide attempts were also recorded throughout the duration of the study period. If present up to the primary endpoint, suicide attempts were classified as serious adverse events and potentially treatment-related.

#### 2.3.3 Preliminary efficacy measures

Preliminary efficacy was evaluated via clinician-rated and self- and parent-reported measures completed at baseline, post-treatment, and at the 3-, 6-, and 12-month follow-ups, unless otherwise indicated.

BDD symptom severity during the past week was measured with the semi-structured, clinician-rated BDD-YBOCS-A. It contains 12 Likert-type items ranging from 0 to 4: five questions on obsessions, five on compulsions, one about insight, and one to measure avoidance behaviour. Total scores range from 0 to 48, with higher scores denoting higher symptom severity. The BDD-YBOCS-A has good internal consistency, adequate convergent and divergent validity, and sensitivity to change (Monzani et al., 2022). Treatment response was defined as a ≥30% reduction on the BDD-YBOCS-A, respective to the baseline score, while full or partial symptom remission was defined as a score ≤16 on the BDD-YBOCS-A (Fernández de la Cruz et al., 2021). The BDD-YBOCS-A was also administered at the 1- and 2-month follow-ups.

The Clinical Global Impairment – Severity Scale (CGI-S) is a single-item global clinician rating of symptom severity, in this case focused on BDD. Scores range from 1 (‘not at all ill’) to 7 (‘among the most extremely ill’) (Guy, 1976). The CGI-Improvement (CGI-I) is a single-item clinician rating of symptom improvement (not administered at baseline). The CGI-I provides a rating of BDD symptom improvement, compared to baseline, with a range from 1 (‘very much improved’) to 7 (‘very much worse’) (Guy, 1976). The CGI-S and the CGI-I have adequate psychometric properties (Busner and Targum, 2007; Leon et al., 1993).

The CGAS is a widely validated clinician-rated measure of the global functioning of a young person during the last month; it comprises one item ranging from 1 (more disabled) to 100 (best functioning). The CGAS has high reliability, as well as discriminant and concurrent validity (Shaffer et al., 1983).

The Appearance Anxiety Inventory (AAI) is a self-reported measure that covers cognitions and behaviours typical of BDD (Veale et al., 2014). It consists of 10 items, with a total score ranging from 0 to 40, where higher scores indicate higher symptom severity. It includes two subscales: avoidance and threat monitoring. The questionnaire has good internal consistency, adequate convergent validity with the clinician-administered BDD-YBOCS-A, and adequate sensitivity to change (Gumpert et al., Under review).

Depressive symptoms were assessed via the child-rated SMFQ-C, the parent-rated SMFQ-P, and the additional suicide item (mentioned in 2.3.2). Both the child and the parent version of the SMFQ include 13 items and have established validity and reliability (Rhew et al., 2010). These questionnaires were additionally administered at the 1- and 2-month follow-ups.

The Work and Social Adjustment Scale – Youth and Parent Versions (WSAS-Y and WSAS-P, respectively) are short instruments assessing functional impairment in five areas: school, daily situations, social activities, leisure activities, and relationships (Jassi et al., 2020b). The total scores range from 0 to 40, with higher scores denoting more impairment. The instruments have excellent psychometric properties, with high internal consistency, good convergent and divergent validity, and sensitivity to change (Jassi et al., 2020b).

The KIDSCREEN-10 – Child (C) and Parent (P) versions (Ravens-Sieberer and Kidscreen Group Europe, 2006) are generic quality of life measures for young people. They consist of 11 items (of which 10 make up an index), each with a 5-level response category. The questionnaire has good psychometric properties (Ravens-Sieberer et al., 2005).

### 2.4 Power calculation

The power calculation was based on an estimated within-group effect size of Cohen's *d* = 1.0 on the BDD-YBOCS-A. The estimation was made based on the results of the only RCT that has evaluated face-to-face CBT for adolescents with BDD (Mataix-Cols et al., 2015). In that trial, the within-group effect size from baseline to the 2-month follow-up was *d* = 1.38. Due to the Internet format, we expected a slightly lower effect size in the current study. The power analysis showed that 16 participants were required to obtain 95% power with an alpha value of 5%. To ensure sufficient power, accounting for a 20% data loss, we aimed to recruit 20 participants.

### 2.5 Statistical analyses

Rates of participant retention, treatment completion and adherence to treatment, treatment acceptability, treatment credibility, treatment satisfaction, and adverse events were summarised descriptively by providing frequencies and percentages, for categorical variables, or means and standard deviations (SD), for continuous variables.

Measures of treatment outcome were analysed through linear mixed models to detect within-group changes over time, following the intention-to-treat principle and a pre-specified analytical plan. To evaluate changes in BDD severity from baseline to the primary endpoint, the first model included all five time points from baseline to the primary endpoint (i.e., baseline, post-treatment, 1-, 2-, and 3-month follow-ups). To investigate treatment durability, a model was fitted including the 3-, 6-, and 12-month follow-up time points. Lastly, a model including all seven time points (baseline to 12-month follow-up) was fitted for graphical representation purposes. Within-group effect sizes were estimated with Cohen's *d* (Cohen, 1988). Statistical significance was set to *p* <0.05 and results were presented with 95% confidence intervals (CI). Treatment response and full or partial remission were calculated at each assessment point from post-treatment and onwards.

## 3 Results

### 3.1 Study participant flow and participant characteristics

**Fig. 1:**
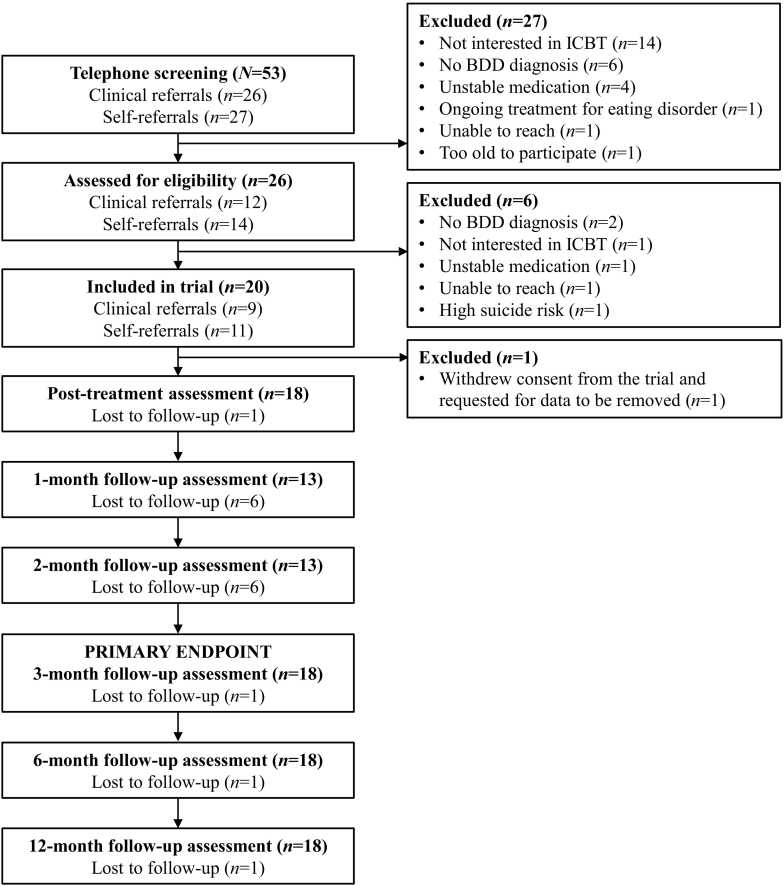
Study participants' flow.

Twenty participants were recruited to the trial between October 20, 2021, and March 17, 2022; the last 12-month follow-up data were collected on June 26, 2023. One participant withdrew consent to participate before the post-treatment time point and requested for their data to be removed from the study. Therefore, all analyses are based on 19 participants (Fig. 1).

As per protocol, participants classified as non-responders after end of the treatment were offered booster video sessions. Two of the five non-responders accepted the offer and received two sessions each between the end of the treatment and the 3-month follow-up. Further, all participants classified as non-responders at the 3-month follow-up were offered additional treatment for BDD at the clinic. Only one participant of five classified as non-responders accepted the offer and then received three face-to-face sessions between the 3-month and the 12-month follow-up.

The sample's demographic and clinical characteristics are presented in Table 1. The sample included 11 self-referred individuals and 8 clinical referrals. Most participants were girls (*n* = 17, 89.5%), with a mean age of 15.6 years (SD = 1.3, range 12–17). Ten (52.6%) adolescents met diagnostic criteria for at least one additional mental disorder, most commonly attention-deficit/hyperactivity disorder or an anxiety disorder. At the start of treatment, seven participants (36.8%) were on pharmacological treatment, mostly SSRIs. The mean BDD-YBOCS-A score at intake was 27.05 (SD = 2.87, range 24–33) and the mean CGI-S score was 4.32 (SD = 2.90), indicating moderately severe BDD symptoms (Table 2).

**Table 1:** Demographic and clinical characteristics of a sample of adolescents with body dysmorphic disorder (*N* = 19).

Clinical characteristics		
	*Mean*	*SD*
Age at assessment	15.8	1.3
Self-reported age of BDD onset	13.4	1.7
	*N*	%

Sex		
Female	17	89.5
Male	2	10.5
Any comorbid mental disorder	10	52.6
Attention-deficit/hyperactivity disorder	6	31.6
Anxiety disorders^a^	3	15.8
Depression	2	10.5
Autism spectrum disorder	1	5.3
On pharmacological treatment	7	36.8
Selective serotonin reuptake inhibitors	4	21.1
Attention-deficit/hyperactivity disorder medication	3	15.8
Melatonin	2	10.5
Antihistamines	1	5.3
Previous psychological treatment for BDD	2	10.5
Family history of obsessive-compulsive related disorders^b^	4	21.1
Poor or absent insight/delusional beliefs^c^	3	15.8
Desire for cosmetic procedure	13	68.4
Any suicidal or self-harm behaviour	8	42.1
Past or current suicide thoughts	6	31.6
Past or current self-harm	7	36.8
CRAFFT - Ever used alcohol	7	36.8
CRAFFT - Ever used other drugs	0	0
School attendance		
Full attendance	12	63.2
Partial attendance	6	31.6
No attendance	1	5.3

*Abbreviations:* BDD, body dysmorphic disorder; CRAFFT, acronym for the key words Car, Relax, Alone, Forget, Friends and Trouble.

a Includes social anxiety disorder, specific phobia, panic disorder or anxiety disorders not otherwise specified.

b Obsessive-compulsive and related disorders in 1st or 2nd degree relatives.

c Defined as 3 or 4 on the insight item of the BDD-YBOCS-A.

**Table 2:** Model estimates for all measures from baseline to the primary endpoint (3-month follow-up) from the linear mixed-effect models.

Measure	M (SE)^a^	Within-group difference	Within-group effect size
		Coefficient (95% CI)^b^	Cohen's *d* (95% CI)^c^
**BDD-YBOCS-A**			
Baseline (*n* = 19)	27.05 (1.35)		
Post (*n* = 18)	14.61 (1.37)	−12.44 (−14.80, −10.08) ⁎⁎⁎	2.29 (1.79, 2.79)
1FU (*n* = 13)	12.52 (1.49)	−14.53 (−17.16, −11.91) ⁎⁎⁎	3.03 (2.31, 3.74)
2FU (*n* = 13)	10.22 (1.49)	−16.83 (−19.46, −14.20) ⁎⁎⁎	4.11 (3.54, 4.68)
3FU (*n* = 18)	11.78 (1.37)	−15.27 (−17.63, −12.91) ⁎⁎⁎	2.94 (2.49, 3.40)
**CGI-S**			
Baseline (*n* = 19)	4.32 (0.20)		
Post (*n* = 18)	2.18 (0.20)	−2.13 (−2.57, −1.70) ⁎⁎⁎	2.58 (2.07, 3.09)
3FU (*n* = 18)	1.96 (0.20)	−2.35 (−2.79, −1.92) ⁎⁎⁎	3.07 (2.57, 3.58)
**CGI-I**			
Post (*n* = 18)	2.22 (0.20)		
3FU (*n* = 18)	2.11 (0.20)	−0.11 (−0.37, 0.15)	0.12 (−,25, 0.50)
**CGAS**			
Baseline (*n* = 19)	52.79 (1.55)		
Post (*n* = 18)	62.75 (1.58)	9.96 (6.97, 12.94) ⁎⁎⁎	1.60 (1.19, 2.02)
3FU (*n* = 18)	63.69 (1.58)	10.90 (7.92, 13.89) ⁎⁎⁎	1.77 (1.34, 2.20)
**AAI**			
Baseline (*n* = 19)	27.89 (1.85)		
Week3 (*n* = 16)	24.41 (1.94)	−3.48 (−6.87, −0.09) ⁎	0.55 (0.17, 0.92)
Week6 (*n* = 15)	22.90 (1.98)	−4.99 (−8.46, −1.53) ⁎⁎	0.73 (0.26, 1.20)
Week9 (*n* = 12)	15.71 (2.10)	−12.18 (−15.92, −8.45) ⁎⁎⁎	1.86 (1.32, 2.40)
Post (*n* = 15)	16.27 (1.97)	−11.62 (−15.08, −8.16) ⁎⁎⁎	1.47 (0.99, 1.95)
3FU (*n* = 14)	13.96 (2.01)	−13.93 (−17.47, −10.40) ⁎⁎⁎	2.01 (1.49, 2.53)
**SMFQ-C**			
Baseline (*n* = 19)	15.11 (1.44)		
Week3 (*n* = 16)	14.08 (1.50)	−1.02 (−3.52, 1.48)	0.14 (−0.18, 0.46)
Week6 (*n* = 15)	14.32 (1.53)	−0.78 (−3.34, 1.77)	0.11 (−0.24, 0.47)
Week9 (*n* = 12)	8.84 (1.61)	−6.26 (−9.13, −3.52) ⁎⁎⁎	1.12 (0.61, 1.63)
Post (*n* = 15)	11.35 (1.53)	−3.75 (−6.30, −1.20) ⁎	0.59 (0.19, 0.99)
1FU (*n* = 13)	7.63 (1.58)	−7.47 (−10.15, −4.79) ⁎⁎⁎	1.36 (0.92, 1.81)
2FU (*n* = 11)	6.39 (1.65)	−8.71 (−11.54, −5.88) ⁎⁎⁎	1.59 (1.06, 2.12)
3FU (*n* = 14)	9.05 (1.55)	−6.06 (−8.66, −3.45) ⁎⁎⁎	1.18 (0.72, 1.64)
**SMFQ-P**			
Baseline (*n* = 19)	11.63 (1.41)		
Week3 (*n* = 17)	11.03 (1.46)	−0.60 (−3.22, 2.02)	0.14 (−0.13, 0.43)
Week6 (*n* = 17)	9.19 (1.46)	−2.44 (−5.07, 0.19)	0.51 (−0.14, 0.87)
Week9 (*n* = 14)	7.73 (1.54)	−3.90 (−6.70, −1.10) ⁎⁎	0.76 (0.37, 1.14)
Post (*n* = 16)	8.99 (1.48)	−2.64 (−5.32, 0.03)	0.42 (−0.03, 0.86)
1FU (*n* = 4)	5.82 (2.36)	−5.81 (−10.3, −1.33) ⁎	1.13 (0.14, 2.12)
2FU (*n* = 11)	5.97 (1.65)	−5.66 (−8.70, −2.63) ⁎⁎⁎	1.06 (0.44, 1.67)
3FU (*n* = 14)	8.95 (1.54)	−2.68 (−5.48, 0.12)	0.50 (0.05, 0.95)
**WSAS-Y**			
Baseline (*n* = 19)	20.37 (1.62)		
Post (*n* = 15)	11.38 (1.77)	−8.99 (−12.45, −5.52) ⁎⁎⁎	1.23 (0.81, 1.65)
3FU (*n* = 14)	9.72 (1.82)	−10.65 (−14.20, −7.09) ⁎⁎⁎	1.56 (1.02, 2.09)
**WSAS-P**			
Baseline (*n* = 19)	19.21 (2.14)		
Post (*n* = 16)	12.70 (2.26)	−6.51 (−10.47, −2.54) ⁎⁎	0.56 (−0.12, 1.23)
3FU (*n* = 14)	11.87 (2.35)	−7.34 (−11.51, −3.18) ⁎⁎	0.84 (0.11, 1.55)
**KIDSCREEN-10-C**			
Baseline (*n* = 19)	30.26 (1.32)		
Post (*n* = 15)	33.10 (1.46)	2.84 (−0.36, 6.04)	0.55 (−0.11, 1.21)
3FU (*n* = 14)	35.50 (1.51)	5.24 (1.96, 8.51) ⁎⁎	0.90 (0.25, 1.55)
**KIDSCREEN-10-P**			
Baseline (*n* = 19)	31.95 (1.34)		
Post (*n* = 16)	34.21 (1.43)	2.26 (−0.59, 5.11)	0.39 (−0.14, 0.93)
3FU (*n* = 14)	35.25 (1.51)	3.31 (0.32, 6.29) ⁎	0.69 (0.20, 1.19)

*Abbreviations:* 1FU, 1-month follow-up; 2FU, 2-month follow-up; 3FU, 3-month follow-up; AAI, Appearance Anxiety Inventory; BDD-YBOCS-A, Yale-Brown Obsessive-Compulsive Scale, modified for BDD – Adolescent version; CGAS, Children's Global Assessment Scale; CGI-S, Clinical Global Impression – Severity; CI, confidence interval; KIDSCREEN-10-C, KIDSCREEN-10 – Child Version; KIDSCREEN-10-P – KIDSCREEN-10 – Parent Version; M, mean; Post, post-treatment; SD, standard deviation; SMFQ-C, Short Mood and Feeling Questionnaire, Child Version; SMFQ-P, Short Mood and Feeling Questionnaire, Parent Version; WSAS-Y, Work, Social and Adjustment Scale–Youth Version; WSAS-P, Work, Social and Adjustment Scale – Parent Version.

⁎ *p* < 0.05.

⁎⁎ *p* < 0.01.

⁎⁎⁎ *p* < 0.001.

a Estimated means and standard errors from the mixed-effects regression model.

b Coefficients at the post-treatment, 1-month, 2-month, and 3-month follow-up compare with the baseline time point.

c Bootstrapped effect sizes (*d*) are derived from the mixed-effects regression model.

### 3.2 Feasibility measures

The time to recruit the 20 initial participants was approximately 20 weeks. We needed to screen 53 potential participants to reach the intended sample size of 20 (1:2.6 ratio). Attrition was low, with 94.7% complete data on the clinician-rated measures at both the primary endpoint and the 12-month follow-up. The average number of completed modules was 7.2 (SD = 4.4) for adolescents and 6.2 (SD = 3.3) for caregivers. Only six adolescents (31.6%) and one caregiver (5.3%) completed all 12 modules.

Clinician-rated adherence to the treatment was acceptable, both at mid- and post-treatment. Three weeks into treatment, both adolescents and caregivers rated the treatment as credible. Treatment satisfaction at the primary endpoint (3-month follow-up) was moderate to high in both adolescents and caregivers (**Supplementary Table 3**).

### 3.3 Safety measures

One participant attempted suicide between baseline and the primary endpoint. The attempt ended in hospitalisation and that participant dropped out of treatment and did not provide any further data. Another participant attempted suicide between the primary endpoint and the 12-month follow-up, but continued to provide follow-up data after the event. Two additional participants reported non-suicidal self-injuries during the study period. All participants and most caregivers reported additional adverse events in the NEQ between baseline and the 3-month follow-up (e.g., stress, hopelessness, conflicts between participant and caretaker). Approximately half of these events were self-reported to be related to the treatment (**Supplementary Table 4**).

### 3.4 Preliminary efficacy at the primary endpoint

Means and standard errors (SEs) from the mixed-effects regression analyses for all measures at each time point are shown in Table 2, while raw means and SDs are shown in **Supplementary Table 2**. The models showed a significant reduction on the BDD-YBOCS-A from baseline to the primary endpoint (3-month follow-up) (coefficient [95% CI] = −15.27 [−17.63 to −12.91], *p* < 0.001). The within-group effect size (Cohen's *d*) was 2.94 (95% CI, 1.98 to 3.85) (Table 2). At the 3-month follow-up, 14 (73.7%) participants were classified as treatment responders and 12 (63.2%) as full or partial remitters.

Mixed-effects regression analyses from baseline to the 3-month follow-up also showed significant reductions on self-reported BDD symptoms (AAI), self-reported depressive symptoms (SMFQ-C), functional impairment (WSAS-Y and WSAS-P), and improved global functioning (CGAS) and quality of life (KIDSCREEN-10-C and KIDSCREEN-10-P) (Table 2).

As per protocol, given to their condition of non-responders when evaluated at post-treatment, two participants received two booster video sessions each before the primary endpoint (3-month follow-up). Additionally, four participants (21.1%) reported protocol deviations between the end of treatment and the primary endpoint as they had received additional care outside the trial (one was hospitalised following a suicide attempt, one received a few supportive sessions for depressive symptoms, one was prescribed fluoxetine for depression, and one stimulant medication for ADHD). In a post-hoc sensitivity analysis, the main model for the BDD-YBOCS-A was repeated excluding all data points after the boosters or protocol deviations for these six participants. The results remained largely unchanged (−15.40 [−18.08 to −12.72], *p* < 0.001).

### 3.5 Preliminary efficacy at the long-term follow-up

**Fig. 2:**
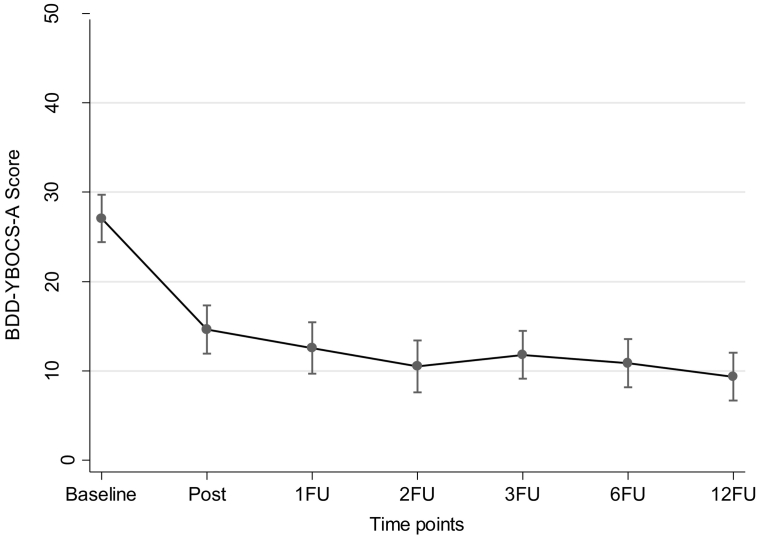
Estimated means on the BDD-YBOCS-A from a mixed-effects regression model including all seven time points (*n* = 19). *Note:* Error bars indicate 95% confidence intervals. *Abbreviations:* 1FU, 1-month follow-up; 2FU, 2-month follow-up; 3FU, 3-month follow-up; 6FU, 6-month follow-up; 12FU, 12-month follow-up; BDD-YBOCS-A, Yale-Brown Obsessive-Compulsive Scale, modified for BDD – Adolescent version; Post, Post-treatment.

Means and SEs from the long-term mixed-effects regression analyses are shown in Table 3, and raw means and SDs in **Supplementary Table 2**. Mixed-effects regression analyses showed a significant further improvement between the primary endpoint (3-month follow-up) and the 12-month follow-up on the BDD-YBOCS-A (−2.44 [−4.23 to −0.66], *p* <0.001). The within-group effect size (Cohen's *d*) for the BDD-YBOCS-A between the 3-month follow-up and the 12-month follow-up was 0.38 (95% CI, 0.06 to 0.70) (Table 3). Fig. 2 depicts the results on the BDD-YBOCS-A from a mixed-effect regression including all study time points. At the 12-month follow-up, 15 (78.9%) participants were classified as treatment responders and 14 (73.7%) were in full or partial remission.

**Table 3:** Model estimates for all measures from the primary endpoint (3-month follow-up) to the 12-month follow-up from the linear mixed-effect model.

Measure	M (SE)^a^	Within-group difference^b^	Within-group effect size
		Coefficient (95% CI)	Cohen's *d* (95% CI)^c^
**BDD-YBOCS-A**			
3FU (*n* = 18)	11.61 (1.47)		
6FU (*n* = 18)	10.67 (1.47)	−0.94 (−2.73, 0.84)	0.14 (−0.03, 0.32)
12FU (*n* = 18)	9.17 (1.47)	−2.44 (−4.23, −0.66) ⁎⁎	0.38 (0.06, 0.70)
**CGI-S**			
3FU (*n* = 18)	1.94 (0.21)		
6FU (*n* = 18)	1.83 (0.12)	−0.11 (−0.46, 0.24)	0.12 (−0.17, 0.41)
12FU (*n* = 18)	1.83 (0.12)	−0.11 (−0.46, 0.24)	0.12 (−0.35, 0.58)
**CGI-I**			
3FU (*n* = 18)	2.11 (0.18)		
6FU (*n* = 18)	2.06 (0.18)	−0.06 (−0.24, 0.13)	0.07 (−0.03, 0.17)
12FU (*n* = 18)	1.78 (0.18)	−0.33 (−0.52, −0.15) ⁎⁎⁎	0.42 (0.14, 0.71)
**CGAS**			
3FU (*n* = 18)	63.83 (2.25)		
6FU (*n* = 18)	61.56 (2.25)	−2.28 (−5.78, 1.23)	−0.25 (−0.54, 0.03)
12FU (*n* = 18)	62.33 (2.25)	−1.50 (−5.01, 2.01)	−0.15 (−0.56, 0.27)
**AAI**			
3FU (*n* = 14)	14.08 (2.02)		
6FU (*n* = 13)	14.31 (2.04)	0.22 (−2.69, 3.14)	−0.01 (−0.22, 0.19)
12FU (*n* *=* 13)	10.79 (2.04)	−3.29 (−6.28, −0.30) ⁎	0.40 (−0.11, 0.90)
**SMFQ-C**			
3FU (*n* = 14)	8.28 (1.66)		
6FU (*n* = 13)	10.35 (1.69)	2.07 (−1.12, 5.25)	−0.33 (−0.83, 0.16)
12FU (*n* = 12)	9.19 (1.74)	0.91 (−2.44, 4.27)	−0.28 (−,87, 0.31)
**SMFQ-P**			
3FU (*n* = 14)	8.20 (1.78)		
6FU (*n* = 15)	8.80 (1.73)	0.60 (−2.99, 4.18)	−0.11 (−0.71, 0.49)
12FU (*n* = 15)	5.57 (1.94)	−2.63 (−6.57, 1.31)	0.48 (−0.11, 1.07)
**WSAS-Y**			
3FU (*n* = 14)	9.19 (1.96)		
6FU (*n* = 13)	11.84 (2.02)	2.65 (−1.67, 6.98)	−0.33 (−0.83, 0.18)
12FU (*n* = 13)	7.75 (2.02)	−1.45 (−5.84, 2.95)	0.17 (−0.57, 0.90)
**WSAS-P**			
3FU (*n* = 14)	11.91 (1.94)		
6FU (*n* = 15)	12.13 (1.90)	0.22 (−3.55, 3.98)	−0.03 (−0.29, 0.23)
12FU (*n* = 15)	7.54 (1.90)	−4.37 (−8.19, −0.54) ⁎	0.55 (−0.05, 1.16)
**KIDSCREEN-10-C**			
3FU (*n* = 14)	35.45 (1.73)		
6FU (*n* = 13)	34.33 (1.77)	−1.11 (−4.49, 2.26)	−0.15 (−0.55, 0.25)
12FU (*n* = 12)	35.07 (1.82)	−0.38 (−3.93, 3.17)	−0.12 (−0.66, 0.42)
**KIDSCREEN-10-P**			
3FU (*n* = 14)	35.97 (1.67)		
6FU (*n* = 15)	35.30 (1.63)	−0.66 (−4.11, 2.79)	−0.19 (−0.68, 0.44)
12FU (*n* = 15)	36.36 (1.63)	0.39 (−3.11, 3.89)	0.10 (−0.47, 0.67)

*Abbreviations:* 3FU, 3-month follow-up; 6FU, 6-month follow-up; 12FU, 12-month follow-up; AAI, Appearance Anxiety Inventory; BDD-YBOCS-A, Yale-Brown Obsessive-Compulsive Scale, modified for BDD – Adolescent version; CGAS, Children's Global Assessment Scale; CGI-S, Clinical Global Impression – Severity; CI, confidence interval; KIDSCREEN-10-C, KIDSCREEN-10 – Child Version; KIDSCREEN-10-P – KIDSCREEN-10 – Parent Version; M, mean; Post, post-treatment; SD, standard deviation; SMFQ-C, Short Mood and Feeling Questionnaire, Child Version; SMFQ-P, Short Mood and Feeling Questionnaire, Parent Version; WSAS-Y, Work, Social and Adjustment Scale–Youth Version; WSAS-P, Work, Social and Adjustment Scale – Parent Version.

⁎ *p* < 0.05.

⁎⁎ *p* < 0.01.

⁎⁎⁎ *p* < 0.001.

a Estimated means and standard errors from the mixed-effects regression model.

b Coefficients at the 6-month and 12-month follow-up compare with the 3-month follow-up time point.

c Bootstrapped effect sizes (*d*) are derived from the mixed-effects regression model.

There was a continued significant improvement between the 3-month and the 12-month follow-up on the AAI, the CGI-I, and the WSAS-P, and results were maintained for the CGI-S, the CGAS, the SMFQ-C, the SMFQ-P, the WSAS-Y, the KIDSCREEN-10-C, and the KIDSCREEN-10-P (Table 3).

Between the 3-month and the 12-month follow-up, 12 (63.2%) participants reported that they had received additional care, most commonly newly prescribed SSRIs, change of SSRI dose or newly prescribed ADHD medication (see **Supplementary Table 5** for details). Due to the large proportion of individuals that received additional interventions during the long-term follow-up, we did not run additional sensitivity analyses.

### 3.6 Therapist support time during the active phase of treatment

The average therapist time per participant per week (children and parents/caregivers combined) was 7.9 min (SD = 4.5, range 3.5–22.3). This included messages in the platform, occasional telephone calls, and the video booster sessions up to the primary endpoint.

## 4 Discussion

We evaluated the feasibility of adapting an existing evidence-based CBT treatment manual for adolescents with BDD (Mataix-Cols et al., 2015) into an Internet-delivered format with brief therapist support. Both the adolescents and their caregivers rated the intervention as credible and satisfactory. Recruitment time was relatively brief, reflecting the shortage of specialist care for this patient group, and attrition was low, in line with previous therapist-guided ICBT trials (Andrén et al., 2022; Aspvall et al., 2021; Nordh et al., 2021). Further, therapist support time was only a fraction of that required in traditional face-to-face CBT (<8 min per participant per week), indicating potential cost-effectiveness. As expected in this patient group (Mataix-Cols et al., 2015; Phillips et al., 2006; Rautio et al., 2022), adverse events were not uncommon, with two suicide attempts during the study period.

ICBT was associated with a significant reduction of BDD symptoms, as measured with the BDD-YBOCS-A, with a large within-group effect size (*d* = 2.94) at the primary endpoint (3-month follow-up). At this time point, about three quarters of our participants were classified as responders and two thirds were in full or partial remission. Although these results are encouraging, is should be noted that a third of the participants received additional treatment outside the standard protocol. However, a post-hoc sensitivity analysis excluding these data points showed that the results remained largely unchanged.

Large improvements were also observed on self-reported BDD symptoms, self-reported depressive symptoms, global functioning, quality of life, and impairment. The gains were not only maintained 12-months after treatment, but both clinician- and self-rated BDD symptom severity continued to improve throughout the follow-up. However, these positive results should be tempered down by the fact that most participants needed and received additional psychological and pharmacological interventions for other comorbid conditions such as depression and ADHD during the follow-up. This illustrates the importance of following up these patients in the long run.

This study had some limitations. Because this was an uncontrolled trial, we cannot conclude that the observed improvements were exclusively due to the evaluated treatment. However, we know that spontaneous remission without treatment is unlikely (Harrison et al., 2016). Still, future RCTs should carefully consider relevant and credible control comparators to test against the active condition (Goldberg et al., 2023). This trial also had a less severe and complex sample, compared to previous studies in young people (Mataix-Cols et al., 2015; Rautio et al., 2022). Therefore, we do not know if ICBT is suitable for more severe and complex cases of BDD. Finally, all participants in this trial were assessed and supported by one single clinical psychologist who is highly specialised in the treatment of BDD.

## 5 Conclusions

ICBT with minimal therapist support is a feasible, preliminary efficacious, and durable treatment alternative for adolescents with BDD. It is important that self-harm and suicidal behaviours, typical of this patient group, are carefully monitored during treatment, regardless of its modality. The evaluation of this intervention in an RCT is warranted.

## Funding

Daniel Rautio was partially funded by a grant from the Martin Rind Foundation.

## Declaration of competing interest

David Mataix-Cols receives royalties for contributing articles to UpToDate, Wolters Kluwer Health. Lorena Fernández de la Cruz receives royalties for contributing articles to UpToDate, Wolters Kluwer Health and for editorial work from Elsevier. All other authors report no conflicts of interest.

## Appendix A Supplementary data

Supplementary material Image 1
