# Supplementary material for: Therapist-guided, Internet-delivered cognitive behaviour therapy for adolescents with body dysmorphic disorder: A feasibility trial with long-term follow-up
Source: Internet Interv. 2023 Nov 16;34:100688. doi: 10.1016/j.invent.2023.100688 (PMC10685040; doi:10.1016/j.invent.2023.100688)

**Online supplementary material**

**ONLINE SUPPLEMENTARY TABLES**

**Supplementary Table 1.** Content of the ICBT modules for adolescents and parents.

| **Modules** | **Youth modules** | **Parent modules** |
| --- | --- | --- |
| **1** | **Introduction and information on BDD**   - What is ICBT for BDD - Overview of the treatment - Information about BDD - Negative thoughts - Rituals | **Introduction and information on BDD**   - What is ICBT for BDD - Overview of the treatment - How the treatment works - Your role as a parent - Information about BDD |
| **2** | **Learn more about BDD**   - Why do you have BDD? - Consequences of BDD - Information about anxiety - Functional analysis of BDD - Goal formulation - Avoidance | **Learn more about BDD**   - Consequences of BDD - How ICBT can help - Goal formulation - Rituals - Avoidance - Family accommodation |
| **3** | **What is exposure?**   - Information on fear and anxiety - Plan your exposure task - Information on habituation - Information on exposure | **What is exposure?**   - Information on fear and anxiety - Plan your exposure task - Information on habituation - Information on exposure |
| **4** | **Start practice exposure**   - Your first exposure task   - Plan   - Execute   - Evaluate - Response prevention | **Help your child with exposure**   - Parental strategies - Help plan an exposure task |
| **5** | **Focus on response prevention**   - Response prevention - BDD and social media - Do’s and don’ts | **Focus on response prevention**   - Obstacles for treatment - Dealing with obstacles - Response prevention - BDD and social media - Do’s and don’ts |
| **6** | **Consequences for families and schools**   - Consequences for the family - Common accommodations - How to limit accommodations - Consequences for school - Information to school - Information on cosmetic procedures - Exposure and response prevention (ERP) tasks | **Consequences for families and schools**   - Consequences for the family - Common accommodations - How to limit accommodations - Consequences for school - Information to school - Information on cosmetic procedures - Exposure and response prevention (ERP) tasks |
| **7** | **BDD thoughts and focus shift**   - Information on focus shift - Practice focus shift - Info on BDD thoughts - Working with negative thoughts - ERP tasks | **BDD thoughts and focus shift**   - Information on focus shift - Practice focus shift - Info on BDD thoughts - Working with negative thoughts - ERP tasks |
| **8** | **Full response prevention**   - Remaining rituals - Examples of full response prevention | **Assist your child in full response prevention**   - Remaining rituals and accommodations - Examples of full response prevention |
| **9** | **More exposure and response prevention**   - Remaining rituals and avoidances | **More exposure and response prevention**   - Remaining rituals and avoidances |
| **10** | **Even more exposure and response prevention**   - Remaining rituals and avoidances | **Even more exposure and response prevention**   - Remaining rituals and avoidances |
| **11** | **Evaluate your goals, start to look forward**   - Challenge your core fear - Planning the future | **Evaluate goals, start to look forward together**   - Challenge your child’s core fear - Planning the future |
| **12** | **Plan ahead**   - Recap of the treatment - What has helped you? - Evaluate your goals - How have your parents helped? - Prepare for setbacks - Plan for future challenges - Evaluate the treatment | **Plan ahead**   - Recap of the treatment - What has helped you? - Evaluate the goals - How have you been able to help your child? - Prepare for setbacks - Plan for future challenges - Evaluate the treatment |

*Abbreviations:* BDD, body dysmorphic disorder; ICBT, Internet-delivered cognitive behaviour therapy.

**Supplementary Table 2.** Raw means and standard deviations for all measures across time points.

| **Measures** | **Baseline** | **Post** | **1FU** | **2FU** | **3FU** | **6FU** | **12FU** |
| --- | --- | --- | --- | --- | --- | --- | --- |
|  | **M (SD)** | **M (SD)** | **M (SD)** | **M (SD)** | **M (SD)** | **M (SD)** | **M (SD)** |
| **BDD-YBOCS-A** | 27.05 (2.90) | 14.44 (7.36) | 13.23 (6.55) | 9.69 (5.98 | 11.61 (6.96) | 10,67 (6.34) | 9.17 (5.86) |
| **CGI-S** | 4.32 (.48) | 2.17 (1.10) | - | - | 1.94 (1.00) | 1.83 (.86) | 1.83 (.92) |
| **CGI-I** | - | 2.22 (1.00) | - | - | 2.11 (.76) | 2.06 (.76) | 1.78 (.81) |
| **CGAS** | 52.79 (3.47) | 62.89 (8.22) | - | - | 63.83 (8.19) | 61.56 (9.42) | 62.33 (11.57) |
| **AAI** | 27.89 (6.67) | 16.00 (10.48) | - | - | 12.79 (8.75) | 13.38 (9.26) | 10.46 (6.80) |
| **SMFQ-C** | 15.11 (5.66) | 11.40 (7.04) | 7.23 (5.20) | 5.73 (5.52) | 7.43 (7.24) | 9.92 (5.89) | 10.17 (6.48) |
| **SMFQ-P** | 11.63 (5.49) | 9.75 (7.74) | 5.25 (5.12) | 6.27 (5.66) | 8.14 (7.64) | 9.07 (7.42) | 7.00 (5.13) |
| **WSAS-Y** | 20.37 (6.83) | 11.40 (7.37) | - | - | 8.86 (7.82) | 11.62 (8.40) | 7.77 (7.36) |
| **WSAS-P** | 19.21 (9.62) | 13.81 (9.63) | - | - | 11.29 (9.17) | 12.20 (8.19) | 7.20 (5.71) |
| **KIDSCREEN-10-C** | 30.26 (3.85) | 33.00 (6.19) | - | - | 35.71 (7.96) | 34.77 (7.42) | 34.08 (4.62) |
| **KIDSCREEN-10-P** | 31.95 (4.56) | 33.94 (7.23) | - | - | 35.86 (6.31) | 35.07 (7.46) | 36.33 (6.44) |
| *Abbreviations:* 1FU, 1-month follow-up; 2FU, 2-month follow-up; 3FU, 3-month follow-up; 6FU, 6-month follow-up; 12FU, 12-month follow-up; AAI, Appearance Anxiety Inventory; BDD-YBOCS-A, Yale-Brown Obsessive-Compulsive Scale, modified for BDD – Adolescent version; CGAS, Children´s Global Assessment Scale; CGI-I, Clinical Global Impression – Impairment; CGI-S, Clinical Global Impression – Severity; KIDSCREEN-10-C, KIDSCREEN-10 – Child Version; KIDSCREEN-10-P – KIDSCREEN-10 – Parent Version; M, mean; Post, post-treatment; SD, standard deviation; SMFQ-C, Short Mood and Feeling Questionnaire, Child Version; SMFQ-P, Short Mood and Feeling Questionnaire, Parent Version; WSAS-Y, Work, Social and Adjustment Scale–Youth Version; WSAS-P, Work, Social and Adjustment Scale – Parent Version. | | | | | | | |

**Supplementary Table 3.** *Treatment credibility*, as measured by a three-item questionnaire, three weeks into treatment (participant, *n*=16; parent, *n*=17); *adherence to treatment*, as measured by the clinician-rated iiPAS at week 6 and post-treatment (*n*=18); and *treatment satisfaction*, as measured with a nine-item questionnaire at post-treatment (participant, *n*=14; parent, *n*=14).

|  | **M (SD)** | **M (SD)** |
| --- | --- | --- |
| **Question and scale – Treatment credibility** | **Participant** | **Parent** |
| How well suited is ICBT for adolescents with BDD? (0 = not at all, 5 = very well) | 3.13 (.72) | 2.88 (1.11) |
| How much improvement do you expect from ICBT? (0 = none, 5 = much) | 2.56 (.73) | 3.18 (.81) |
| How motivated are you for ICBT? (0 = not at all, 5 = very motivated) | 2.63 (.96) | 3.47 (0.87) |
| **Question and scale – Treatment adherence** | **Week 6** | **Post** |
| Work pace (0 = totally inactive, 5 = works at full pace) | 3.00 (1.14) | 2.72 (1.27) |
| Engagement in treatment tasks (0 = no tasks done, 5 = all tasks done) | 2.94 (1.06) | 2.78 (1.44) |
| Communication with therapist (0 = no communication, 5 = active communication) | 2.56 (1.29) | 2.44 (1.46) |
| Motivation to change (0 = uses no strategies, 5 = active use of strategies) | 2.94 (1.11) | 3.22 (1.11) |
| How frequent the patient logs in (0 = never logs in, 5 = logs in often) | 2.28 (1.31) | 2.67 (1.37) |
| **Question and scale – Treatment satisfaction** | **Participant** | **Parent** |
| How has the treatment helped with BDD? (0 = not at all, 5 = very much) | 2.64 (1.22) | 2.86 (.95) |
| How convenient was the internet format for you? (0 = not at all, 5 = very convenient) | 2.79 (1.05) | 3.21 (1.19) |
| How difficult was it to work through the modules? (0 = very difficult, 5 = very easy) | 2.21 (.80) | 2.29 (1.27) |
| How difficult was it to write to the therapist? (0 = very difficult, 5 = very easy) | 3.29 (.99) | 3.71 (.47) |
| How difficult was it to understand the tasks? (0 = very difficult, 5 = very easy) | 2.71 (.83) | 2.93 (1.14) |
| Would you recommend the treatment to a friend? (0 = definitely not, 5 = definitely) | 3.00 (1.24) | 3.29 (1.20) |
| Do you think the treatment provided enough help? (0 = definitely not, 5 = definitely) | 2.36 (1.28) | 2.29 (1.20) |
| In sum, what did you think about the treatment? (0 = really bad, 5 = really good) | 2.79 (.89) | 3.29 (1.07) |
| What format would you have preferred? (0 = F2F, 1 = doesn’t matter, 2 = ICBT) | 1.00 (.78) | 1.07 (.92) |
| *Abbreviations:* BDD, body dysmorphic disorder; ICBT, Internet-delivered cognitive behaviour therapy; iiPAS, internet intervention Patient Adherence Scale; M, mean; SD, standard deviation. | | |

**Supplementary Table 4.** Self-reported adverse events at mid-treatment, post-treatment and 3-month follow-up, according to the NEQ.

|  | **Self-report** | | **Parent-report** | |
| --- | --- | --- | --- | --- |
| **Adverse event from baseline to mid-treatment (*n*=15)** | ***n*** | **Assessed as related to ICBT**  ***n*** | ***n*** | **Assessed as related to ICBT**  ***n*** |
| Sleeping problems | 7 | 7 | 4 | 2 |
| Stress | 10 | 7 | 8 | 4 |
| Anxiety or fear | 8 | 4 | 4 | 2 |
| Worry | 10 | 5 | 4 | 2 |
| Hopelessness | 9 | 7 | 5 | 2 |
| Feelings of discomfort | 8 | 5 | 3 | 0 |
| Worsening of BDD problems | 6 | 3 | 3 | 1 |
| Thinking more of unpleasant memories | 2 | 1 | 3 | 1 |
| Fear of people knowing about participation in treatment | 5 | 1 | 2 | 2 |
| Suicide thoughts | 2 | 0 | 2 | 2 |
| Embarrassment of participating in treatment | 1 | 0 | 0 | 0 |
| Stopped believing that things could be better | 9 | 9 | 5 | 3 |
| Started to believe that BDD symptoms could not improve | 6 | 2 | 7 | 3 |
| Becoming depended on the treatment | 1 | 0 | 0 | 0 |
| Could not always understand the treatment | 8 | 2 | 6 | 2 |
| Lack of confidence in treatment | 5 | 1 | 3 | 0 |
| Feeling that the treatment did not help | 10 | 5 | 8 | 4 |
| Lack of motivation | 5 | 2 | 3 | 1 |
| Conflicts between adolescent and primary caretaker | 8 | 6 | 6 | 2 |
| **Total number of adverse events reported** | **120** | **67** | **76** | **33** |
|  | **Self-report** | | **Parent-report** | |
| **Adverse event from mid-treatment to post-treatment (*n*=15)** | ***n*** | **Assessed as related to ICBT**  ***n*** | ***n*** | **Assessed as related to ICBT**  ***n*** |
| Sleeping problems | 4 | 4 | 3 | 2 |
| Stress | 6 | 5 | 5 | 2 |
| Anxiety or fear | 5 | 3 | 3 | 2 |
| Worry | 4 | 3 | 3 | 2 |
| Hopelessness | 3 | 3 | 2 | 2 |
| Feelings of discomfort | 2 | 0 | 6 | 2 |
| Worsening of BDD problems | 2 | 1 | 1 | 1 |
| Thinking more of unpleasant memories | 3 | 2 | 4 | 1 |
| Fear of people knowing about participation in treatment | 1 | 0 | 2 | 0 |
| Suicide thoughts | 2 | 2 | 3 | 1 |
| Embarrassment of participating in treatment | 1 | 0 | 1 | 0 |
| Stopped believing that things could be better | 6 | 5 | 6 | 4 |
| Started to believe that BDD symptoms could not improve | 4 | 3 | 5 | 3 |
| Becoming depended on the treatment | 0 | 0 | 1 | 0 |
| Could not always understand the treatment | 7 | 3 | 5 | 1 |
| Lack of confidence in treatment | 6 | 2 | 4 | 1 |
| Feeling that the treatment did not help | 5 | 3 | 6 | 1 |
| Lack of motivation | 10 | 2 | 5 | 2 |
| Conflicts between adolescent and primary caretaker | 6 | 6 | 7 | 3 |
| **Total number of adverse events reported** | **77** | **47** | **72** | **30** |
|  | **Self-report** | | **Parent-report** | |
| **Adverse event from post-treatment to 3-month follow-up (*n*=14)** | ***n*** | **Assessed as related to ICBT**  ***n*** | ***n*** | **Assessed as related to ICBT**  ***n*** |
| Sleeping problems | 2 | 2 | 2 | 2 |
| Stress | 8 | 4 | 5 | 3 |
| Anxiety or fear | 5 | 3 | 6 | 4 |
| Worry | 0 | 0 | 2 | 2 |
| Hopelessness | 3 | 2 | 3 | 2 |
| Feelings of discomfort | 3 | 2 | 5 | 2 |
| Worsening of BDD problems | 3 | 2 | 2 | 1 |
| Thinking more of unpleasant memories | 3 | 2 | 1 | 1 |
| Fear of people knowing about participation in treatment | 1 | 0 | 1 | 0 |
| Suicide thoughts | 4 | 4 | 1 | 1 |
| Embarrassment of participating in treatment | 2 | 1 | 1 | 0 |
| Stopped believing that things could be better | 7 | 6 | 2 | 1 |
| Started to believe that BDD symptoms could not improve | 5 | 3 | 1 | 0 |
| Becoming depended on the treatment | 0 | 0 | 1 | 0 |
| Could not always understand the treatment | 5 | 0 | 4 | 0 |
| Lack of confidence in treatment | 3 | 2 | 0 | 0 |
| Feeling that the treatment did not help | 5 | 2 | 4 | 1 |
| Lack of motivation | 3 | 0 | 4 | 1 |
| Conflicts between adolescent and primary caretaker | 4 | 3 | 4 | 1 |
| **Total number of adverse events reported** | **66** | **38** | **49** | **22** |
| *Abbreviations:* BDD, body dysmorphic disorder; ICBT, Internet-delivered cognitive behaviour therapy; NEQ, Negative Effects Questionnaire. | | | | |

**Supplementary Table 5.** Additional care between the 3-month follow-up and the 12-month follow-up.

| **Type of additional care** | ***n*** |
| --- | --- |
| Newly prescribed SSRI for anxiety and/or depression | 2 |
| Newly prescribed SSRI and received ICBT for depression | 1 |
| Newly prescribed SSRI and medication for ADHD | 1 |
| Changed dose of SSRI | 2 |
| Changed dose of SSRI and received duloxetine for anxiety | 1 |
| Changed dose of SSRI and received a few supportive sessions for depression | 1 |
| Newly prescribed ADHD medication | 1 |
| Received a few supportive sessions for depression | 1 |
| Assessed for, and received a few supportive sessions, for eating disorder | 2 |
| **Any kind of additional care** | **12** |
| *Abbreviations:* ADHD, attention-deficit/hyperactivity disorder; SSRI, selective serotonin reuptake inhibitors. | |

**ONLINE SUPPLEMENTARY FIGURES**

**Supplementary Figure 1.** Screenshots from Internet-delivered treatment for adolescents with body dysmorphic disorder.

1. Overview and start page, with list of modules.


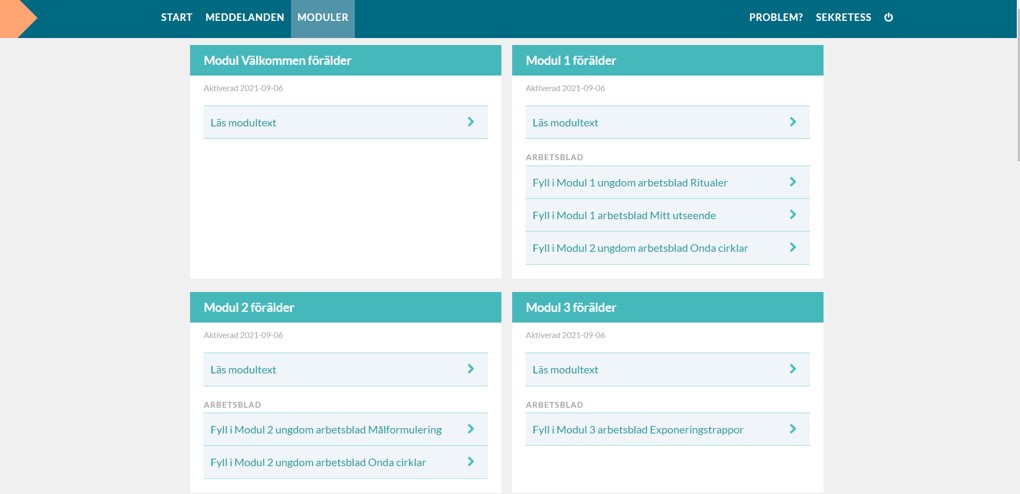


2. Educational film, used for delivering psychoeducation and the treatment rationale.


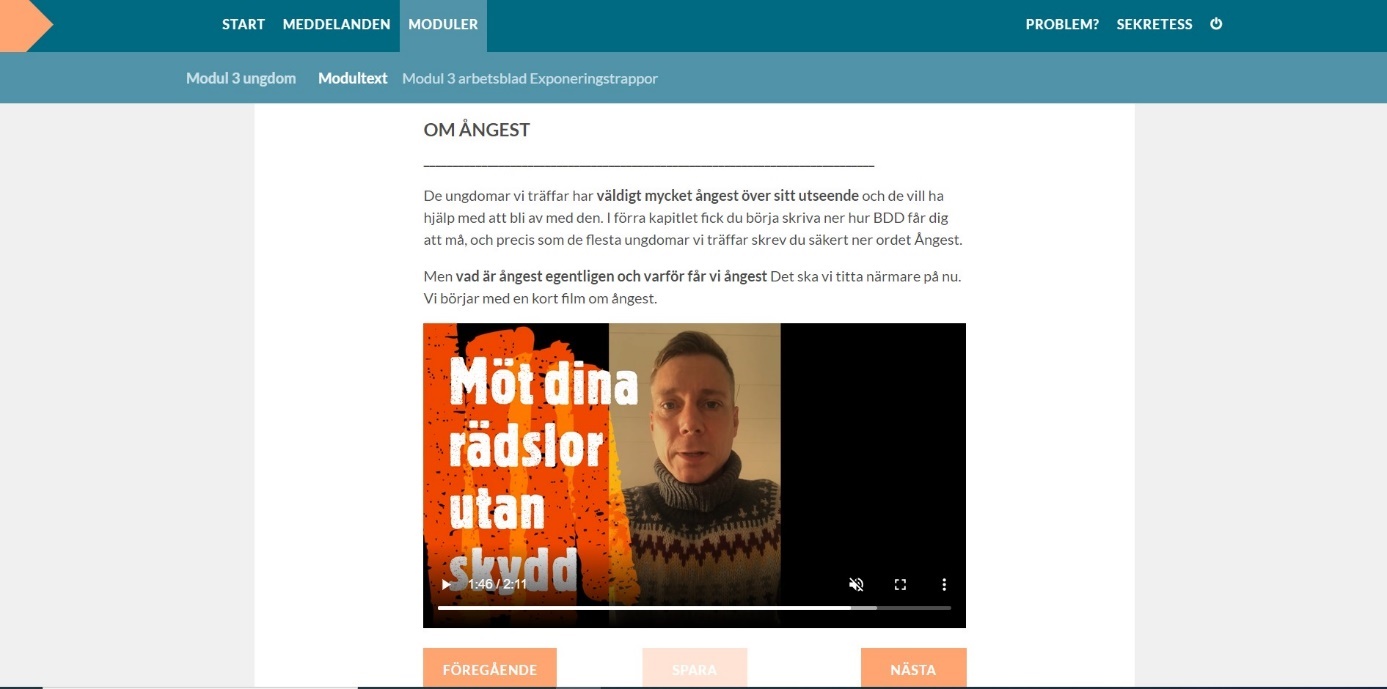


3. Psychoeducation on anxiety and habituation.


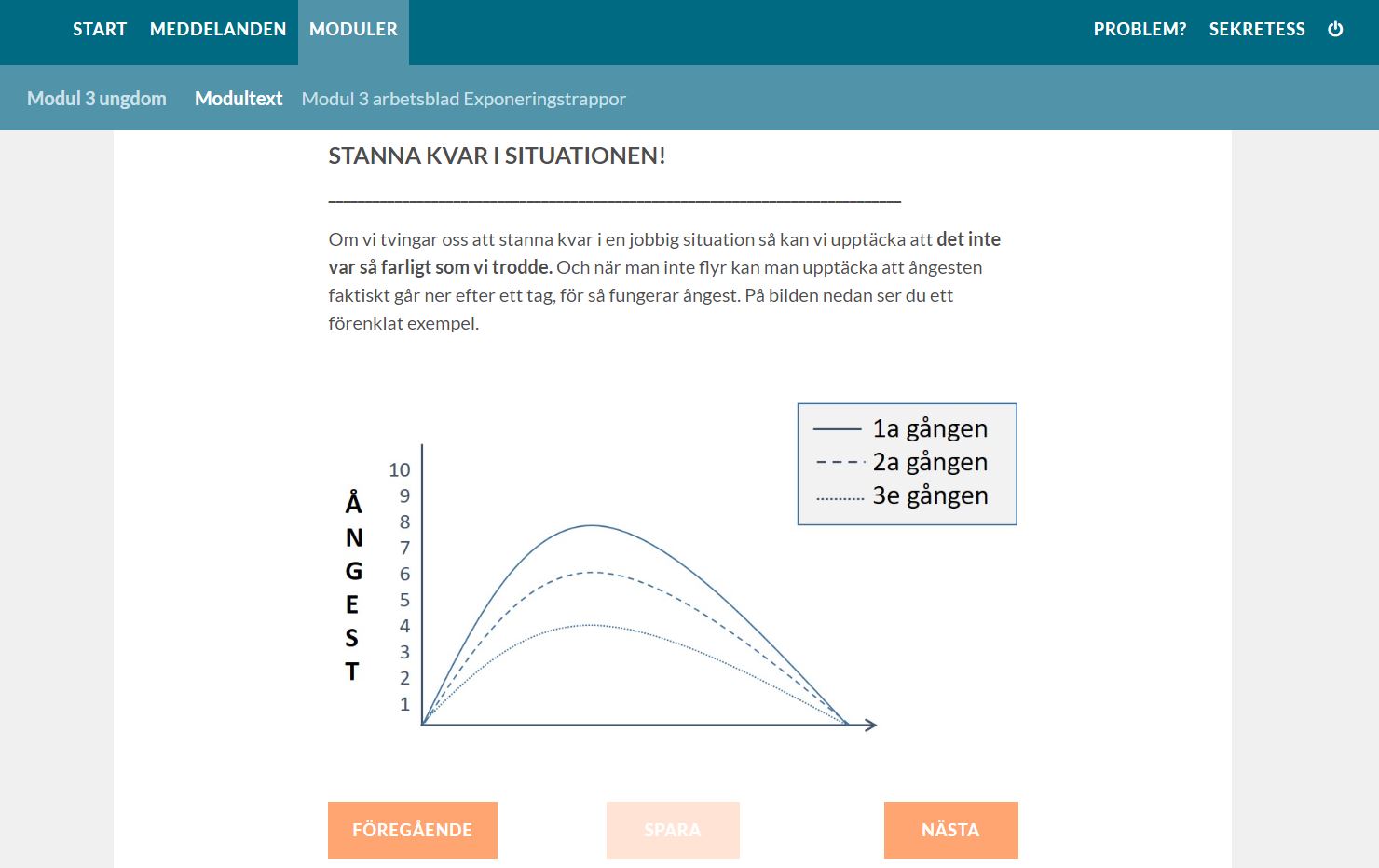


4. Psychoeducation on functional analysis of body dysmorphic disorder symptoms.


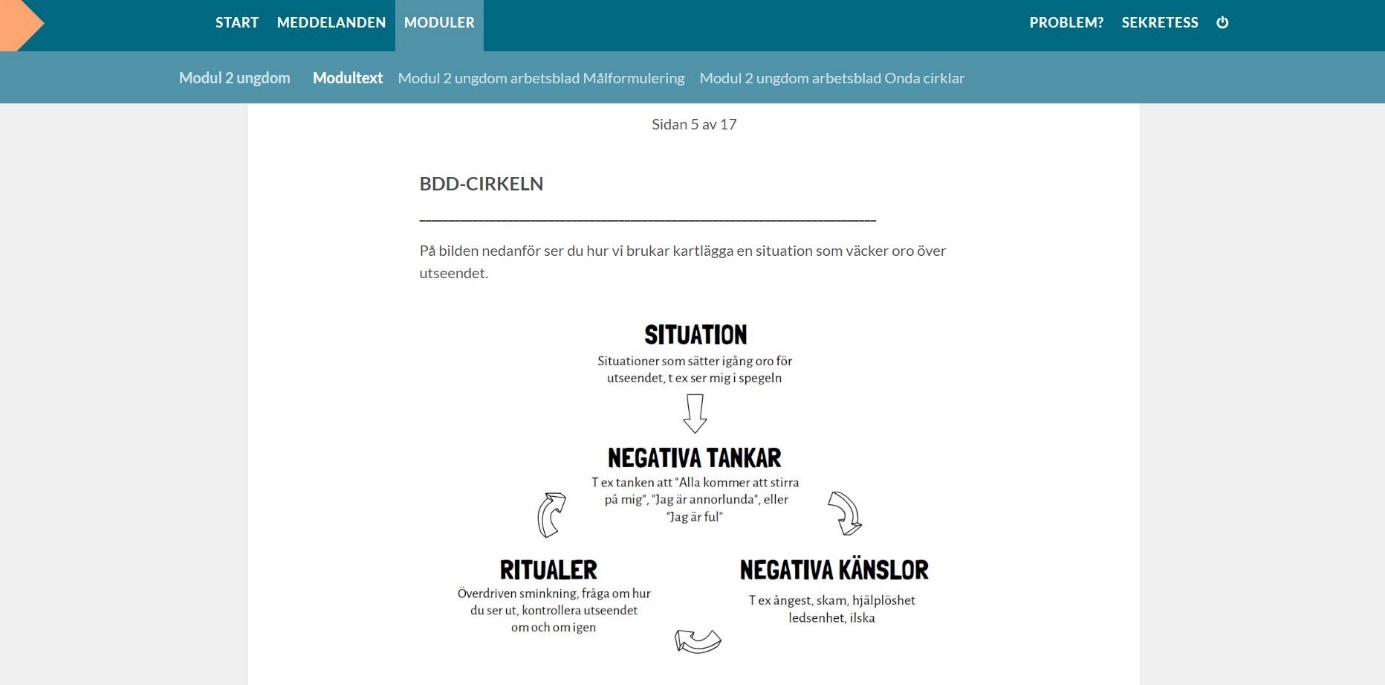

Supplement: Supplementary file 1 — Supplementary material [file mmc1.docx]
